# Is the future scarless? – Fibroblasts as targets for scarless wound healing: a narrative review

**DOI:** 10.1177/20595131221095348

**Published:** 2022-09-01

**Authors:** Dylan Parry, Keith Allison

**Affiliations:** 1 12186 Newcastle University Medical School, Newcastle upon Tyne, UK; 2 South Tees Hospitals NHS Foundation Trust, Middlesbrough, UK

**Keywords:** Scarless, fibroblast, Engrailed-1, TGFβ, miRNA, siRNA

## Abstract

**Introduction:** Scarless healing is the ideal outcome of wound healing and is exhibited in some species. This narrative review assembles the current understanding of fibroblast heterogenicity along with the latest fibroblast-related targets for scar reduction therapies. Human regenerative wound healing is deemed possible due to the wound regeneration already seen in the early gestation foetus. **Methods:** This literature narrative review was undertaken by searching PubMed and Web of Science databases and Google Scholar to find articles concerning the fibroblast involvement in wound healing. We evaluated and collated these articles to form a consensus of the current understanding of the field. **Discussion:** This article describes current understanding of fibroblast heterogenicity and involvement in wound healing, focusing on the role of fibroblasts during physiological scarring. We also present the current most promising targets involving fibroblasts in the reduction of scarring and how we can manipulate the behaviour of fibroblasts to mimic the wound regeneration models in the human foetus. These targets include the pro-fibrotic EN1 positive fibroblast lineage, TGFβ1 inhibition, and genetic therapies utilising miRNAs and siRNAs. **Conclusion:** No therapies are currently available to eradicate scarring; however, treatment options are available to reduce the appearance of scarring. Further research into the heterogenicity and interactions of fibroblasts in both the foetus and adult is needed, and this may lead to the development of novel treatments against scarring.

**Lay Summary:**

Scarless healing refers to the repair of a wound with minimal residual scarring. The main cell responsible for the repair process is the fibroblast. It is now understood that there are different types of fibroblasts. Simply, some of these fibroblasts lead to scarring and some lead to regeneration. The early human foetus has mainly regenerative fibroblasts, but during aging the number of scarring fibroblasts increase to become the majority in the adult . Understanding how we can modify this process may ultimately result in the reduction in scarring. Currently, scar reduction therapies are aimed at optimal wound healing, surgical removal of abnormal scars, and using steroids and other drugs to encourage better wound repair by limiting the effect of scarring fibroblasts. Future therapies aim to target specific groups of fibroblasts to encourage regenerative wound healing. This narrative review aims to cover the current understanding of the different groups of fibroblasts and their effect on wound healing. We also cover the current and potential therapies that can be used to reduce scarring and suggest further areas for research in this field.

## Introduction

Fibroblasts are mesenchymal cells that account for the majority of the cellular density of the dermis and have a crucial role in wound healing.^1^ Until recently, fibroblasts were not considered to have extensive involvement in the field of scarless wound healing and were seen only as extracellular matrix (ECM) producing cells.^1^ It is now understood that there are many lineages of human fibroblasts with distinct and heterogeneous functions. The discovery of this heterogeneity has led to an increased focus on the role of different fibroblast lineages in skin regeneration and scarring.

Scarring is the typical physiological outcome of wound healing. It is an evolutionary adaptation that provides quick and effective repair to damaged tissues, sometimes at the expense of tissue integrity and function. Scar tissue lacks skin appendages and has an organised collagen structure replacing the typical “basket-weave” dermal structure in unwounded tissues, leading to reduced tensile strength. Ideal wound repair would involve regeneration of the normal skin structure, including its associated appendages.

The ability to prevent scarring has applications beyond cosmetic and aesthetic uses, with the ability to restore function to extensively damaged tissues and preclude pathological scarring. Understanding the fundamental (patho)physiology of fibrosis that underpins scarring may ultimately translate to the development of novel therapies and treatments for use in clinical practice.

## Fibroblasts and scarring

### Fibroblast lineages

Only recently there has been the discovery of pan-fibroblast surface markers in human fibroblasts in all dermal layers. These surface markers are platelet-derived growth factor receptor (PDGFR) alpha, PDGFR beta and Cluster of Differentiation 90 (CD90).^2–4^ These markers can be used to identify and differentiate fibroblasts from other cell types in human tissue samples, however, they are not perfect fibroblast markers since they are neither 100% sensitive nor specific.

Mapping fibroblast lineages has proven difficult due to progeny not remaining attached to one another and the lack of sub-population surface marker identification. Lineage tracing in mice has been carried out and has identified that a common fibroblast progenitor gives rise to dermal papilla fibroblasts and papillary and reticular dermal fibroblast progenitors.^5^ Two dermal fibroblast lineages have been identified by lineage tracing and transplantation assays, one forming the upper dermis (papillary fibroblasts, dermal papilla fibroblasts and arrector pili muscles) and the other forming the lower dermis (reticular fibroblasts, pre-adipocytes, adipocytes).^5^ Using comparative spatial and single-cell transcriptional profiling, it has also been further noted that there are at least four distinct murine dermal fibroblast lineages: CD26^+^Sca1^−^ papillary fibroblasts, Dlk1^+^Sca1^−^ reticular fibroblasts, and two pre-adipocyte sub-populations, Dlk1^+^Sca1^+^ and Dlk1^−^Sca1^+^.^3^

Recently, surface markers for human papillary and reticular dermal fibroblast subpopulations have been identified. It has been shown that papillary fibroblasts express FAP^+^CD90^−^ and reticular fibroblasts express FAP^−^CD90^+^, allowing the identification of these sub-populations.^6^ Further research is needed to confirm whether papillary and reticular fibroblasts are distinct lineages or if there is any dynamic change between the two subpopulations.^7^

**Figure 1.**
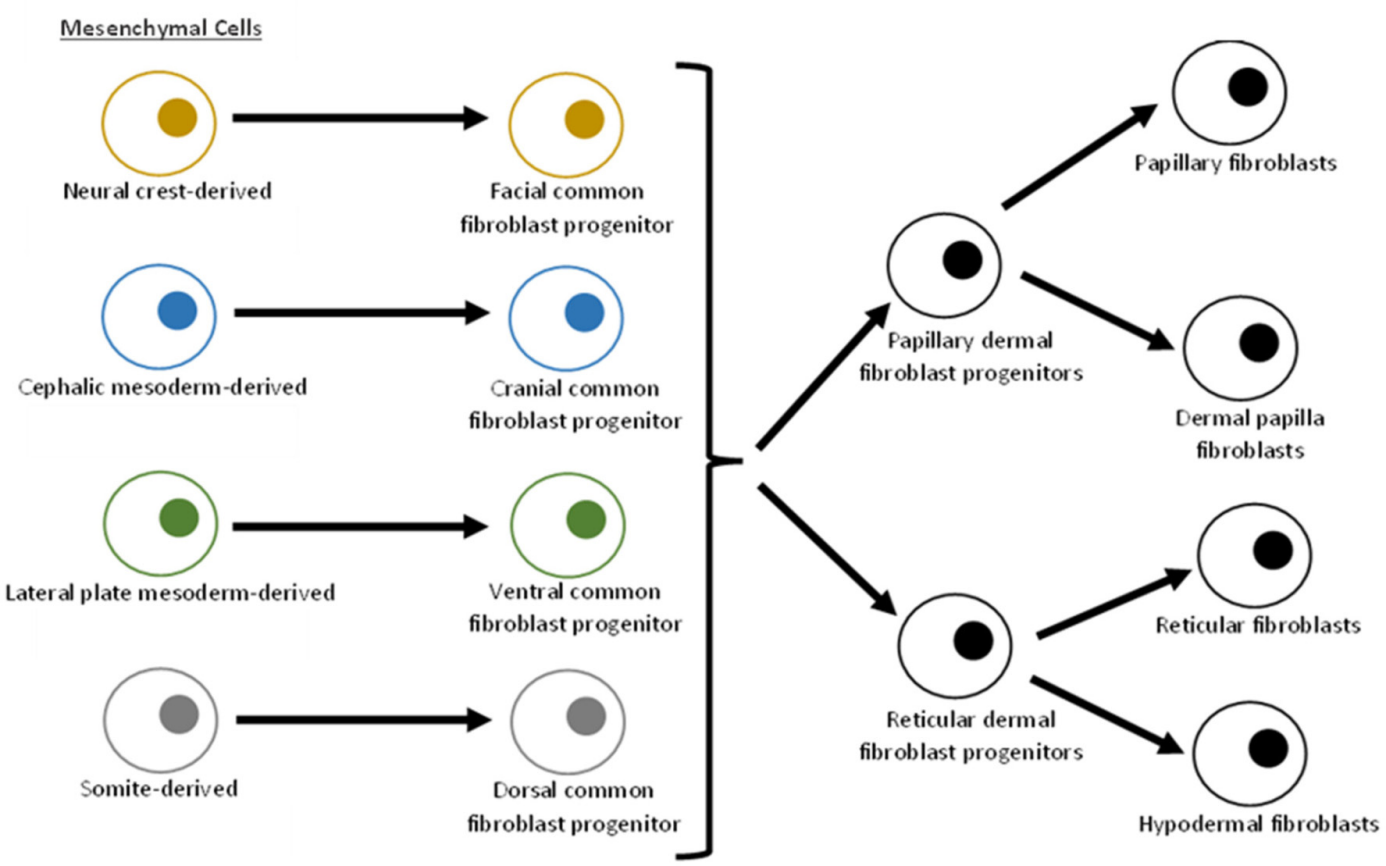
Fibroblast development lineages, representing the development from regional mesenchymal cells to site-specific fibroblast sub-populations.

Cell fate mapping analysis of murine embryos has shown that different anatomical locations of dermal fibroblasts have different embryological origins.^8^ The facial dermal fibroblast lineage is derived from cranial neural crest cells. The cranial dermal fibroblast lineage is derived from the cephalic mesoderm. The dorsal dermal fibroblast lineage is derived from the somite, and the ventral dermal fibroblast lineage is derived from the lateral plate mesoderm.^8^ It is believed that this anatomical distribution of fibroblasts is maintained among other vertebrates, including humans, and fibroblast fate is mediated by the Wnt/β-catenin signalling pathway (Figure 1).^9^

**Figure 2.**
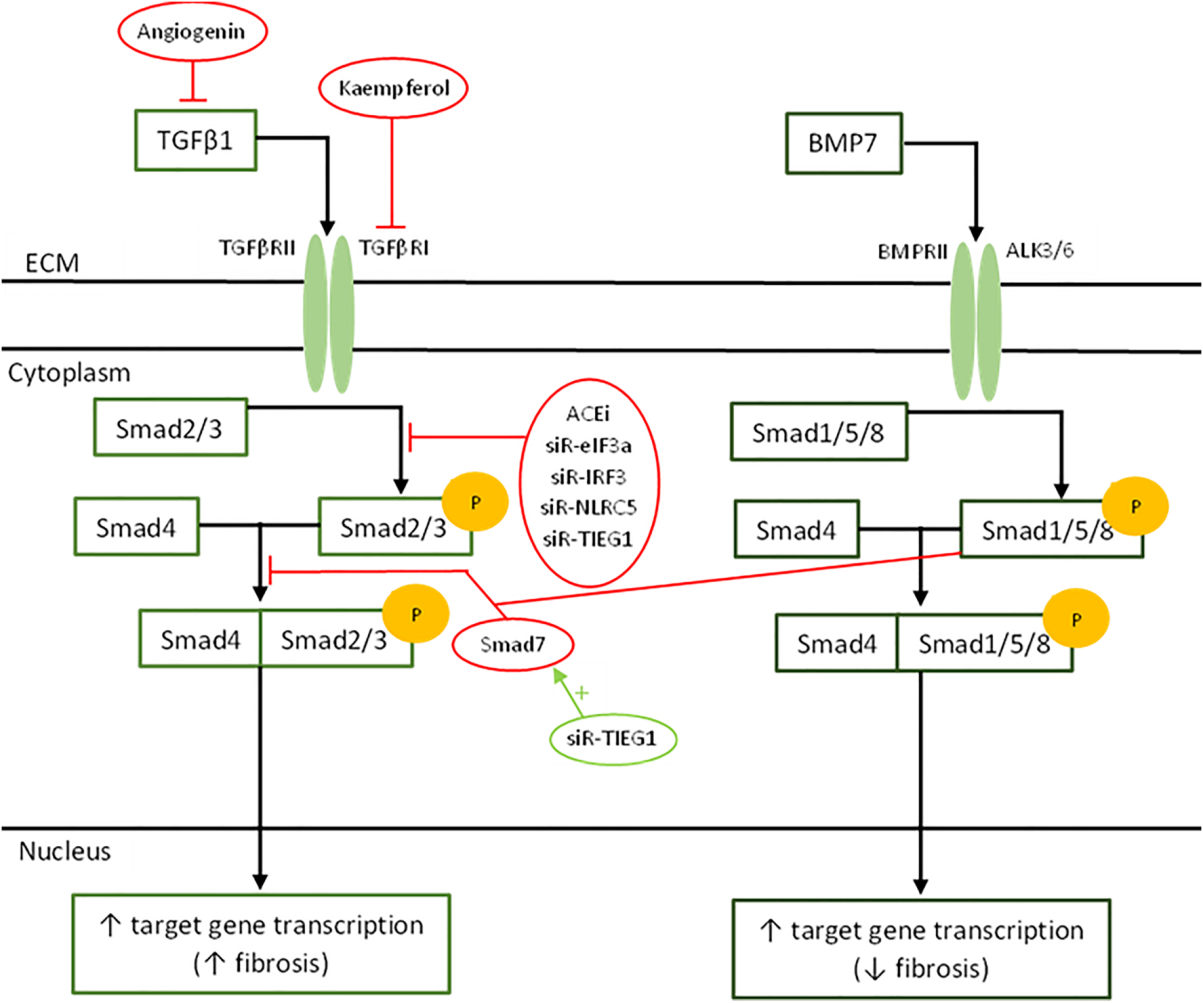
Schematic representation of cellular messaging within fibroblasts during normal wound healing and examples of molecular targets in the reduction of scarring (circled).

Further, embryological fibroblast lineages have been discovered in the murine dermis, which is also believed to be present in humans. The *engrailed-1* (EN1) homeobox gene can be transiently expressed in some fibroblasts during embryological development, creating two distinct lineages present in the dorsal dermis: EN1-lineage-positive fibroblasts (EPFs) and EN1-lineage-negative fibroblasts (ENFs).^10^ EPFs are of great interest in the field of scarless healing as they are pro-fibrotic in nature and replace ENFs, which are regenerative in nature, during embryonic development.^10^ 90% of EPFs are identifiable by their CD26/DPP4 surface marker expression, although it is essential to note that cell surface marker expression is inconsistent between *in vitro* and *in vivo*. The *paired related homeobox gene-1* (Prrx1) can similarly be transiently expressed, contributing another two distinct lineages present in the ventral dermis: Prrx1-lineage-positive fibroblasts (PPFs) and Prrx1-lineage-negative fibroblasts (PNFs).^11^ PPFs are located mainly within dermal perivascular and hair follicle niches and rapidly proliferate upon cutaneous injury.^12^ PPFs and PNFs have synonymous roles with EPFs and ENFs, respectively.^11^ Finally, the Wnt1 gene forms fibroblast lineages in the oral mucosa dermis. Conversely, cells derived from the Wnt1 lineage display a minimal fibrotic phenotype compared to their EPF and PPF counterparts, which could partly account for the more regenerative nature of the oral mucosa during wound healing.^13^

Upon reciprocal transplantation of murine oral and dorsal dermal fibroblasts, it was noted that scar formation was observed in the oral cavity, and a reduction in scarring was observed in the dorsum.^13^ These findings suggest there is intrinsic positional memory among different fibroblast populations, which functions independently from the microenvironment. Hox genes establish this intrinsic positional memory during embryonic development, however, the exact mechanism behind this is yet to be discovered and is an area for further research.^14^

Myofibroblasts can be considered as differentiated and specialised fibroblasts present during wound healing.^15^ Myofibroblasts are derived mainly from reticular fibroblasts upon entry to the wound site.^5^ There is some heterogenicity seen among myofibroblasts, although the current understanding of this heterogenicity is lacking, and further investigation into the origins and sub-populations is needed. However, myofibroblasts may have different activation states based on alpha-smooth muscle actin (α-SMA) expression. α-SMA is the contractile stress fibre found in myofibroblasts, and hence immunostaining for this can help identify these cells in culture.^16^ It is also important to note that myofibroblasts not only originate from fibroblasts but can also arise from epithelial cells, fibrocytes, pericytes and smooth muscle cells.^17,18^

### Fibroblast involvement in wound healing

Fibroblasts were initially thought of as homogenous cells, and the discovery of fibroblast heterogenicity has led to increased interest in the roles of fibroblasts during wound healing. Fibroblasts are now seen as the core cell type during wound healing, which interact with their environment to repair damaged tissues.^19^

Papillary and reticular fibroblasts are spatially and functionally distinct dermal sub-populations implicated in wound healing physiology.^20^ Papillary fibroblasts support epithelium stratification, regulate hair follicle formation, cause weak collagen alignment, and contain little α-SMA.^20^ On the other hand, reticular fibroblasts cause strong collagen alignment, inhibit hair follicle formation, possess abundant α-SMA, and demonstrate differentiation into adipocytes.^20^ This leads to the consensus that papillary fibroblasts have a regenerative nature, whereas reticular fibroblasts have a fibrotic nature.^5^ Wounding triggers an early rapid influx of reticular fibroblasts to the wound site, encouraging a fibrotic response to wound repair.^5^

Embryological fibroblast lineages are a current focal point in the understanding of wound healing, especially with regards to wound regeneration. As previously mentioned, fibroblasts are embryologically derived from different embryological tissues.^8^ The current understanding is that these embryological origins give rise to distinct fibroblast lineages present in different anatomical locations. These lineages then, generally, give rise to fibroblasts with either a pro-regenerative or pro-fibrotic phenotype.^10^ Using flow cytometry analysis, it has been shown that during embryological development, the pro-regenerative lineage-negative fibroblasts are replaced by the pro-fibrotic lineage-positive fibroblasts.^10^ Post-wounding, there is activation of *EN1* in ENFs of the reticular dermis, mediated by activation of the Yes-associated protein (YAP) signalling pathway in response to mechanical wound tension.^21,22^ This increase in the density of EPFs contributes around 40–50% of the fibroblasts at the wound site, which mediates a subsequent fibrotic response.^21^ Proliferation of the PPF subpopulation is also seen at the wound site, contributing to the fibrotic wound healing response.^12^

Myofibroblasts are absent in the unwounded dermis and arise from fibroblast differentiation in the regenerative stage of wound healing.^23^ The significant roles of myofibroblasts are to synthesise and secrete collagen and cause wound contraction to reduce the size of the wound, however, this contraction leads to increased scarring due to the alignment of collagen fibres.^24^ Wound contraction is made possible since myofibroblasts express α-SMA, a contractile filament present in the cell.^25^ Myofibroblast differentiation is due to mechanical wound tension, macrophage activation and molecular signalling with TFGβ1 and TGFβ2 within the wound site.^26^ Once the requirement of myofibroblasts is no longer needed, there is either de-differentiation back into fibroblasts or apoptosis, leading to the cessation of wound remodelling.^27,28^ Over-activation or prolonged activation of myofibroblasts leads to pathological scarring and contractures.^29^

Molecular signalling influences fibroblasts during wound repair involving growth factors and cytokines within the wound environment and intracellular signalling pathways. Transforming growth factor beta (TGFβ) is a group of critical cytokines in the physiology of wound healing; they are secreted by immune cells in early wound healing and by fibroblasts themselves in later wound healing.^30^ In the early embryo, the TGFβ1:TGFβ3 ratio during wound healing is lower than post-natal wound healing.^31^ The action of TGFβ1 causes enhanced pro-fibrotic response, whereas TGFβ3 produces a reciprocal response and may go some way to explain the regenerative nature of embryonic wound healing.^32^ TGFβ1 binds to TGFβRI/II, which upon activation, phosphorylates the Smad2/3 signalling pathway in fibroblasts, leading to the expression of pro-fibrotic genes .^32^ *In vitro* cultured foetal fibroblasts were stimulated with TGFβ1, causing a more myofibroblastic phenotype and subsequently producing an enhanced fibrotic response.^33^ There is also interest in the Wnt/β-catenin signalling pathway, where epidermal activation triggers Sonic hedgehog (Shh) expression, which has been shown to cause proliferation of and ECM remodelling by papillary fibroblasts in the murine dermis.^34^ More recently, non-coding RNAs (e.g. miRNAs and lncRNAs) have proven to be implicated in the regulation of fibroblasts during wound healing.^35^ Many non-coding RNAs have been identified which are implicated in scarring and regeneration of healing wounds by targeting a myriad of signalling pathways involved during wound repair. Pro-fibrotic miRNAs include miR-21, miR-192, miR-141-3p, miR-181a, miR-205 and miR-130a.^36–39^ Moreover, anti-fibrotic miRNAs include miR-29b, miR-98, miR-519d, miR-495, miR-637, miR-1224 and miR-145-5p (Table 1).^38,40–44^

## Therapies for scarless healing via fibroblasts

### Inspiration for scar reduction therapies

Inspiration for scarless healing comes from the natural wound regeneration model during early human embryological development.^45^ The early embryo dermal fibroblasts have majority ENF instead of majority EPF in the adult dermis.^10^ Further, during wound repair, a reduced inflammatory response is seen in the early embryo compared to the adult, which may account for the lower TGFβ1:TGFβ3 ratio seen in the embryo.^46,47^ By investigating the different cellular actions and interactions throughout development and across different anatomical locations, there will be an enhanced understanding of the scarring process and will encourage the innovation of novel therapies in scar reduction.

**Table 1.** Summary of the pro- and anti-fibrotic factors affecting fibroblasts during wound healing (non-exhaustive list).

Pro-fibrotic Factors	Anti-fibrotic factors
TGFβ1	TGFβ3
TGFβ2	miR29b
miR-21	miR-98
miR-192	miR-519d
miR-141-3p	miR-495
miR-181a	miR-637
miR-205	miR-1224
miR-130a	miR-145-5p
Wnt/β-catenin (fibroblasts)	Wnt/β-catenin (keratinocytes)

### Fibroblast-related targets in scar reduction

Most recently, there has been a focus on *EN1*-lineage fibroblasts and their involvement in the process of scar formation. It has been shown that *EN1* is activated in ENFs in response to mechanical wound tension via the YAP signalling pathway.^21^ Inhibition of YAP via verteporfin has been shown to block *EN1* activation in ENFs and promotes ENF-mediated regenerative wound repair, suggesting that post-natal ENFs retain their pro-regenerative ability.^21^ A recent study showed that verteporfin YAP inhibition reduced the expression of pro-fibrotic genes and inhibited the action of TGFβ from inducing actin stress fibres in dermal control fibroblasts.^48^ Further, verteporfin reduced the pro-fibrotic phenotype of fibroblasts in patients with diffuse cutaneous systemic sclerosis. The effect of verteporfin was also demonstrated to be dose-dependent, using concentrations at 0.1 and 1.0 µg/mL.^48^ Hence, verteporfin has proven to be a potentially effective therapy in the treatment and prevention of fibrosis.

The surface marker CD26 is expressed by fibroblast subpopulations implicated in the process of scarring by producing the majority of collagen during wound healing.^49^ The majority of EPFs also expresses CD26; hence this surface marker is a potential target for scar reduction therapy.^50^ Sitagliptin is a potential inhibitor of CD26. A recent *in vitro* study showed that sitagliptin significantly reduced CD26 and type 1 collagen expression, inhibited migration, and promoted apoptosis, suggesting encouraging potential as a scar reduction treatment.^51^ Furthermore, *in* vivo CD26 inhibition with MK0626 in murine wounds led to an increased rate of wound closure and a decrease in scarring, both outcomes of which are of significant clinical interest and benefit.^50^

TGFβ has long been a target for scar reduction therapies. The TGFβ3 recombinant avotermin has previously undergone phase I/II double-blind, randomised control trials where it has been shown to reduce scarring significantly and is safe and well-tolerated.^52–54^ Unfortunately, since these initial trials, the efficacy of avotermin in phase III clinical trials was deemed insufficient.^55^ During these phase III trials, a different standard was set, and hence a 50% lower dose was administered, which may account for the insufficient efficacy.^55^

TGFβ1 inhibition is an additional target for reduction in scarring therapies, and there have been many therapeutic agents used to target the action of the TGFβ1/Smad2/3 signalling pathway in scar fibroblasts. Application of scar fibroblasts with recombinant angiogenin *in vitro* resulted in decreased TGFβ1 secretion and inhibited the TGFβ1/Smad2 signalling pathway, leading to a reduction in fibroblast proliferation and attenuated scarring.^56^ However, the safety of angiogenin is ambiguous due to its role in tumorigenesis.^57^

Intralesional corticosteroids, such as triamcinolone, are commonly used in practice to treat pathological scarring. Corticosteroid mechanism includes suppression of inflammation, reduction of fibroblast proliferation, and cause vasoconstriction leading to reduced delivery of oxygen and nutrients to the wound site.^58^ Intralesional triamcinolone has been shown to have a variable response, with 50–100% regression of the scar, however they have a 50% recurrence rate after 5 years.^59^ Intralesional immunomodulators are also used in scar reduction. 5-fluorouracil (5-FU) is an immunomodulator which works by inhibiting DNA synthesis in rapidly proliferating cells, reducing proliferation of fibroblasts.^60^

In a recent study, intralesional bleomycin has also been shown to be highly effective in reducing hypertrophic and keloid scarring, with a mean reduction in scar volume of 75.85% after 12 months.^61^ The safety of bleomycin has been well established previously for other dermatological indications.^62^ The mechanism of bleomycin is poorly understood but has been shown to inhibit TGFβ-stimulated collagen synthesis.^63^

Baicalein treatment of hypertrophic scars exhibits inhibition of fibroblast proliferation via the TGFβ1/Smad2/3 signalling pathway *in vitro* and *in vivo* and further inhibits α-SMA expression in fibroblasts.^64^ Bone morphogenic protein-7 (BMP-7) is another treatment targeting the TGFβ1/Smad2/3 signalling pathway. BMP-7 has been proven to inhibit TGFβ-induced fibrosis via activation of the inhibitory BMP-7/Smad1/5/8 signalling pathway.^65^ In addition, BMP-7 also induced apoptosis of fibroblasts and increased the expression of α-SMA, CTGF and types 1 and 3 collagen.^65^

Interestingly, the utilisation of ACE inhibitors (ACEi) as an anti-scarring therapy has recently been investigated. ACEi has been shown to inhibit Smad2/3 phosphorylation and reduce TGFβ1 expression, leading to reduced fibroblast proliferation.^66^ Finally, kaempferol can target the TGFβRI on fibroblasts and exert an anti-fibrotic effect by TGFβ1 antagonism.^67^ Kaempferol has been shown to inhibit hypertrophic scarring by inhibiting collagen synthesis and fibroblast proliferation.^67^

Genetic therapies also have a role in regulating the TGFβ/Smad and other signalling pathways present during wound healing. Exogenous small interfering RNAs (siRNAs) have been investigated to knockdown genes associated with fibrosis via interference of the complementary mRNA. siR-eIF3a, siR-IRF3 and siR-NLRC5 have been shown to inhibit TGFβ1 induced keloid fibroblast proliferation, the expression of α-SMA, type 1 collagen, TGFβRI and TGFβRII, and also inhibit the phosphorylation of Smad2/3 in keloid fibroblasts.^68–70^ siR-TGFβRI has been shown to decrease the phosphorylation of Smad2/3, decrease the expression of CTGF, α-SMA, types 1 and 3 collagen, and reduces fibroblast proliferation.^71^ There was also the advantage of requiring a lower dose of siR-TGFβRI as it is more target-specific, reducing its side effects, making it more suitable for clinical use than standard TGFβRI inhibitors.^71^ Finally, siR-TIEG1 reduced the expression of types 1 and 3 collagen, inhibited the TGFβ/Smad2 pathway, but also increased the level of Smad7.^72^ siRNAs have proven to have great potential in treating pathological scarring; however, further research is needed to elicit their efficacy in clinical practice (Figure 2).

Micro RNAs (miRNAs) are able to post-transcriptionally degrade and inhibit target mRNA and hence are critical regulators of gene transcription. Identification of miRNAs that regulate scarring during wound healing will ultimately lead to potential scar reduction therapies. The use of stem cell-derived exosomes has been reported to promote wound regeneration and has been shown to produce a wound repair response similar to that in the early gestation foetus.^73^ Adipocyte-derived stem cell exosomes contain the miRNAs: miR-21, miR-23a, miR-125b, and miR-145, and the application of these exosomes has been shown to reduce scar formation and accelerate wound healing.^74^ Additionally, umbilical cord-derived mesenchymal stem cell-derived exosomes enriched with miR-21, miR-23a, miR-125b, and miR-145 have been demonstrated to reduce scar formation.^75^ The use of these exosomes at the wound site was shown to inhibit the fibrotic response, enhance angiogenesis, stimulate endogenous stem cell recruitment and proliferation, reduce the inflammatory response, and decrease the expression of α-SMA.^75^ The use of stem cell-derived exosomes in clinical practice has great potential as a therapy for scar reduction since the reduction in scarring and accelerated wound repair are both clinically attractive outcomes.

## Conclusions

Currently, there are no treatment options that can eradicate scarring and promote regenerative wound healing in humans. There are, however, many treatment options which can reduce the appearance of scarring, especially in pathological scarring. This review has explained the current understanding of fibroblast heterogenicity, which is still in its infancy and also the involvement of fibroblasts during wound healing, including their molecular regulatory mechanisms. Further research into the heterogenicity of fibroblasts is required, with an enhanced understanding of the fate selection and regulation pathways. This may identify subpopulations of fibroblasts which share common cellular markers which can be targeted in scar reduction therapies, as shown by the recent identification of *EN1* involvement in fibroblasts. Additional work also needs to be done to identify the interactions between different fibroblast subpopulations and their interactions within their niche during wound repair.

Although great advancements have been made in this field, further work needs to be undertaken to fully understand the physiology of wound healing, which will aid in the development of future scarless healing therapies. Also, by gaining further insight into the genetic regulation of wound healing, personalised therapy in the reduction of scarring may become a future possibility. Finally, comprehending the fundamental differences between early foetal and adult wound healing may lead to the development of more novel therapies by modifying adult wound healing to undergo a more foetal-like phenotype. This review has collated the current understanding of fibroblast involvement in wound healing and covered some of the current and novel fibroblast-related therapies with the aim of scar reduction. Our hope is that this review will act as a summary of the current understanding with suggestions for further areas of work to inspire the creation of novel therapies.
